# Theoretical Accuracy of Along-Track Displacement Measurements from Multiple-Aperture Interferometry (MAI)

**DOI:** 10.3390/s140917703

**Published:** 2014-09-23

**Authors:** Hyung-Sup Jung, Won-Jin Lee, Lei Zhang

**Affiliations:** 1 Department of Geoinformatics, The University of Seoul, 90 Jeonnong-dong, Dongdaemun-gu, Seoul 130-743, Korea; E-Mail: twother@uos.ac.kr; 2 The Department of Land Surveying and Geo-Informatics, The Hong Kong Polytechnic University, Kowloon, Hong Kong, China; E-Mail: lslzhang@polyu.edu.hk

**Keywords:** multiple-aperture interferometry (MAI), along-track displacement, synthetic aperture radar (SAR), SAR interferometry (InSAR)

## Abstract

The measurement of precise along-track displacements has been made with the multiple-aperture interferometry (MAI). The empirical accuracies of the MAI measurements are about 6.3 and 3.57 cm for ERS and ALOS data, respectively. However, the estimated empirical accuracies cannot be generalized to any interferometric pair because they largely depend on the processing parameters and coherence of the used SAR data. A theoretical formula is given to calculate an expected MAI measurement accuracy according to the system and processing parameters and interferometric coherence. In this paper, we have investigated the expected MAI measurement accuracy on the basis of the theoretical formula for the existing X-, C- and L-band satellite SAR systems. The similarity between the expected and empirical MAI measurement accuracies has been tested as well. The expected accuracies of about 2–3 cm and 3–4 cm (γ = 0.8) are calculated for the X- and L-band SAR systems, respectively. For the C-band systems, the expected accuracy of Radarsat-2 ultra-fine is about 3–4 cm and that of Sentinel-1 IW is about 27 cm (γ = 0.8). The results indicate that the expected MAI measurement accuracy of a given interferometric pair can be easily calculated by using the theoretical formula.

## 1. Introduction

Synthetic aperture radar (SAR) interferometry (InSAR) has been successfully used for observations of surface displacements such as earthquakes, volcanic eruptions, ground subsidence, landslides, *etc.* [1–11]. Source geometry in ground displacement modeling can be ambiguous if one-dimensional (1D) displacements are only used for the modeling, and hence three-dimensional (3D) displacement fields are strongly required to improve the estimation of the displacement model parameters for volcanic activities, earthquakes, and other processes. However, the InSAR method is limited to measuring 1D displacement measurement along the radar antenna's line-of-sight (LOS) direction.

Many researchers have investigated the measurement of three-dimensional surface displacements from the InSAR method. The multiple radar passes have been used to determine the ground range and vertical components of surface displacements [12,13], but could not be used to measure the along-track displacement. Thus the approach cannot estimate displacements along the N-S direction (approximately along-track direction) because currently available SAR data are acquired by near-polar orbiting satellites. The pixel-offset method has been proposed for measuring the along-track displacement from two or more amplitude SAR images. This method has been widely used for measuring the 3D displacements [14], but it is not allowed to the improvement of the displacement model parameter estimation due to its reduced sensitivity. Consequently, the precise measurement of the along-track displacement is a key issue in measuring the 3D displacements from InSAR method.

A substantial improvement in measuring the along-track displacement has been made with the multiple aperture SAR interferometry (MAI) proposed by Bechor and Zebker [15]. This method can measure the along-track displacement with a measurement accuracy of several centimeters. This method creates forward- and backward-looking interferograms using azimuth sub-aperture processing, and then constructs a MAI interferogram from the two interferograms. Jung *et al.* [16,17] have proposed a further improved method of the MAI processing. Jung's MAI method is designed to reduce interferometric phase noises and correct phase contributions from the flat-Earth and topographic effects. They have revealed that the phase contributions result from the perpendicular baseline difference between forward- and backward-looking interferograms. This MAI method has been successfully used to retrieve 3D surface displacement fields by integrating InSAR and MAI methods [4,18]. In addition, the method has been used to correct ionospheric distortion from a SAR interferogram [19–21].

The empirical accuracy of the MAI measurement has been investigated and compared with that of pixel-offset measurement. The empirical accuracy of the pixel-offset measurement is limited to 12 to 15 cm in high coherent areas for ERS interferometric pairs [15]. The accuracy corresponds to about 2.4%–3.0% of the azimuth resolution, which is about 5.0 m for the ERS SAR images. The pixel offset measurement for the ALOS PALSAR interferometric pair has the improved accuracy of about 7.1 cm [22]. It corresponds to about 1.6% of the azimuth resolution. The empirical accuracies of the MAI measurements for the ERS and ALOS data are about 6.3 cm and 3.57 cm [4,17], respectively, which correspond to only 1.3% and 0.8% of the azimuth resolution, respectively. It demonstrates that the accuracy of MAI measurement is approximately 1.0% of the azimuth resolution as well as two times more accurate than that of pixel-offset tracking measurement. When we measure along-track surface displacements using the MAI method, it is very necessary to expect the measurement accuracy of the MAI method in a given interferometric pair. However, the estimated empirical accuracy cannot be generalized to any interferometric pairs because it largely depends on the system and processing parameters and interferometric coherence used for the accuracy assessment. A theoretical formula for the MAI method is given to expect MAI measurement uncertainties according to the system and processing parameters and interferometric coherence [16]. It means that the expected MAI measurement accuracy can be calculated by using the theoretical formula from the system and processing parameters and coherence of a given interferometric pair.

In this paper, we investigate the expected MAI measurement accuracy on the basis of the theoretical formula for the existing X-, C- and L-band satellite SAR systems and represent the similarity between the expected and empirical MAI measurement accuracies, where the empirical accuracies were calculated by using in-situ GPS measurements. For the investigation, we describe an efficient way to calculate the expected MAI measurement accuracy by considering the coherence, the processed bandwidth, the effective number of looks, noise reduction factor, *etc.* Moreover, we compare the expected MAI measurement accuracies among the existing satellite SAR systems including TerraSAR-X, COSMO-SkyMed, Kompsat-5, ERS-1/2, Envisat, Sentinel-1, Radarsat-2, JERS-1, ALOS PALSAR, ALOS2-PALSAR2.

## 2. Methodology

### 2.1. Review of MAI (Multiple-Aperture Interferometry) Method

Previous researches have proposed MAI methods to measure along-track displacements [15–17]. Using azimuth sub-aperture processing, the methods make forward- and backward-looking SLC images from SAR raw data with the modification of the Doppler Centroid and the reduction of the Doppler bandwidth by one half. Forward- and backward-looking interferograms are obtained from forward- and backward-looking SLC images of master and slave data. A MAI interferogram is produced by complex conjugate multiplication of the forward-looking interferogram with the backward-looking one [15–17]. Finally, phase distortions on the MAI interferogram are corrected.

**Figure 1.**
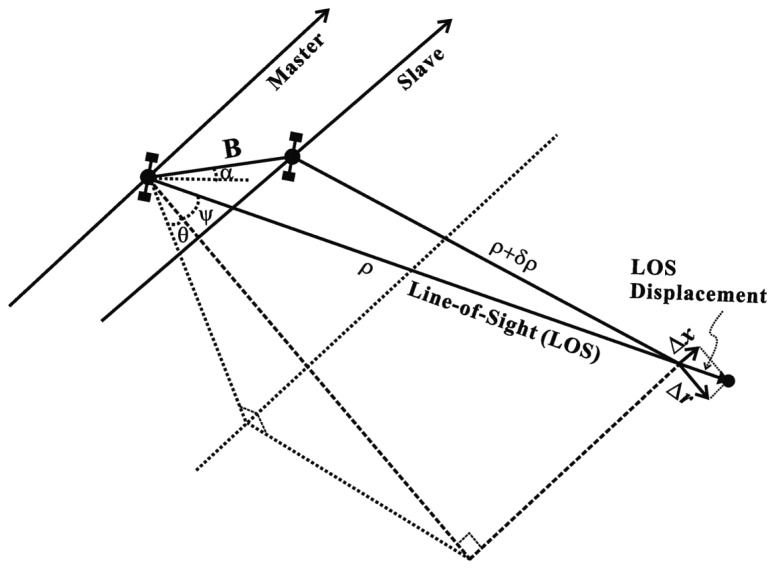
Interferometric imaging geometry, where Δ*r* and Δ*x* are respectively the across- and along-track displacements, and *δρ* is the slant range distance differences between master and slave images.

As shown in Figure 1, the interferometric phase *ϕ_InSAR_* can be defined by the line-of-sight (LOS) displacement, which is composed of across-track displacement (Δ*r*) and along-track displacement (Δ*x*), and the slant range distance differences (*δρ*) between master and slave images as given by:

(1) $$\phi_{\text{InSAR}}=\frac{4\pi }{\lambda }\left(\delta \rho +\Delta r\cos\psi +\Delta x\sin\psi \right)$$

where *λ* is the radar wavelength and *ψ* is squint angle. The *δρ* is approximated to [21]:

(2) δρ≈−B⋅sin(θ−α)

where *θ* and *α* are look angle and baseline orientation, respectively, and *B* is the baseline between the master and slave orbits. By applying the approximation of *ψ* ≈ 0 due to zero Doppler geometry, the InSAR interferometric phase can be rewritten by:

(3) $$\phi_{\text{InSAR}}=-\frac{4\pi }{\lambda }B\cdot \sin(\theta -\alpha )+\frac{4\pi }{\lambda }\Delta r$$

For deriving the MAI phase, we need to define the forward -looking interferometric phase (*ϕ*_*InSAR*,*f*_) as defined by:

(4) $$\phi_{\text{InSAR},f}=-\frac{4\pi }{\lambda }\left(B-\frac{\Delta B}{2}\right)\cdot \sin\left(\theta -\alpha -\frac{\Delta \theta -\Delta \alpha }{2}\right)+\frac{4\pi }{\lambda }\left[\Delta r\cos\left(\psi +\frac{\Delta \beta }{2}\right)+\Delta x\sin\left(\psi +\frac{\Delta \beta }{2}\right)\right]$$

where Δ*B*, Δ*θ*, Δ*α*, and Δ*β* are the differences of the baseline, look angle, baseline orientation, and, antenna angular beam width, respectively, between the forward- and backward-looking pair. Similarly, the backward-looking interferometric phase (*ϕ*_*InSAR*,*b*_) is represented by:

(5) $$\phi_{\text{InSAR},b}=-\frac{4\pi }{\lambda }\left(B+\frac{\Delta B}{2}\right)\cdot \sin\left(\theta -\alpha +\frac{\Delta \theta -\Delta \alpha }{2}\right)+\frac{4\pi }{\lambda }\left[\Delta r\cos\left(\psi -\frac{\Delta \beta }{2}\right)+\Delta x\sin\left(\psi -\frac{\Delta \beta }{2}\right)\right]$$

By applying the approximations of *ψ* ≈ 0 and |Δ*θ* − Δ*α*| ≈ 0 [16], the MAI phase (*ϕ_MAI_*) is defined by:

(6) $$\begin{array}{ll} \phi_{\text{MAI}} & =\phi_{\text{InSAR},f}-\phi_{\text{InSAR},b}\\ & \approx \frac{4\pi }{\lambda }\cdot \Delta B\cdot \sin(\theta -\alpha )+\frac{4\pi }{\lambda }\cdot \Delta \beta \cdot \Delta x \end{array}$$

The antenna angular beam width difference between the forward- and backward-looking pair Δ*β* is:

(7) $$\Delta \beta =n\cdot \beta \approx n\frac{\lambda }{l}$$

where *n* is a normalized squint that presents a fraction of the full aperture width, *β* is the effective antenna beam width, and *l* is the effective azimuth antenna dimension. Consequently, the MAI phase is rewritten as:

(8) $$\phi_{\text{MAI}}=\frac{4\pi }{\lambda }\cdot \Delta B\cdot \sin(\theta -\alpha )+\frac{4\pi }{l}n\Delta x$$

From Equations (3) and (8), it is obvious that the flat-Earth and topographic phases of the InSAR phase are caused by the baseline between master and slave images [21] while those of the MAI phase are caused by the baseline difference between the forward- and backward-looking pairs [16].

The flat-Earth phase difference Δ_*φMAI*,*f*_ from near to far ranges can be defined by [16]:

(9) $$\Delta \phi_{\text{MAI},f}\approx \frac{4\pi }{\lambda }\cdot \frac{\Delta B_{⊥}}{\rho \cdot \tan\theta }\Delta \rho$$

where Δ*B*_⊥_ is the perpendicular baseline which is defined by Δ*B*_⊥_ = Δ*B* ⋅ cos(*θ* − *α*), Δ*ρ* is the slant range distance difference from near to far ranges. Given the C-band SAR system parameters of ERS-1/2 and Envisat, the flat-Earth phase difference from near to far ranges is approximately 140 degree when Δ*B*_⊥_ is 0.1 m. Supposing that a MAI interferogram is processed with *n* = 0.5 and a resulting fringe (360 degree) of 10 m, this flat-Earth phase difference corresponds to the apparent along-track displacement of 3.8 m [16]. And also given the advanced X- and L-band SAR systems of TerraSAR-X and ALOS, the flat-Earth phase difference from near to far ranges is, respectively, approximately 90 and 20 degree when Δ*B*_⊥_ is 0.1 m. Since one fringe is presented by 4.8 and 8.9 m on TerraSAR-X and ALOS PALSAR MAI inteferograms processed with *n* = 0.5, the corresponding along-track displacements are about 1.2 and 0.5 m, respectively. Although neither of the phase distortions are significant compared with conventional cross-track interferometry, they should be corrected in order to improve the measurement accuracy of along-track displacement. Moreover, in the case of conventional interferometry, the flat-Earth phase distortion cannot be perfectly removed because of the atmospheric artifact, while the flat-Earth phase distortion of a MAI interferogram can be removed using the second order polynomial model because it does not possess the atmospheric artifact [16].

The topographic difference Δ*ϕ*_*MAI*,*topo*_ after the flat-Earth correction is also defined as [16]:

(10) $$\Delta \phi_{\text{MAI},\text{topo}}\approx \frac{4\pi }{\lambda }\cdot \frac{\Delta B_{⊥}}{\rho \cdot \sin\theta }\Delta h$$

where Δ*h* is the topographic height. Given the C-band SAR system parameters of ERS-1/2 and Envisat, a topographic difference of 2000 m contributes 7.5 deg. to the MAI phase when Δ*B*⊥ is 0.1 m. And also given the advanced X- and L-band SAR systems of TerraSAR-X and ALOS, the topographic phase difference by a topographic difference of 2000 m is approximately 1.0 and 11.0 degree in TerraSAR-X and ALOS PALSAR systems, respectively, when Δ*B*⊥ is 0.1 m. The topographic phase distortions correspond to the along-track displacements of about 0.21, 0.02 and 0.15 m for ERS-1/2 or Envisat, TerraSAR-X and ALOS PALSAR systems, respectively. The topographic effect is much smaller than the effect of flat-Earth; consequently, the reduction of topographic phase distortion can be optional, depending on the topographic characteristics of the study area, especially for the TerraSAR-X system. And the squint angle difference between the forward- and backward-looking images also leads to mis-registration according to topographic height, which results in a residual topographic phase [16]. This value is generally larger than the predicted topographic phase due to the perpendicular baseline difference. The phase contribution is linearly proportional to topographic height and can be properly removed by using the first order polynomial [16].

After the flat-Earth and topographic phase of the MAI interferogram is corrected, the along-track displacement is defined from the MAI phase as given by:

(11) $$\Delta x=\frac{\phi_{\text{MAI}}}{4\pi }\frac{l}{n}$$

The along-track displacement depends on the MAI phase and the effective azimuth antenna length. Given the ERS-1/2 or Envisat C-band SAR system parameters, one fringe of the InSAR phase represents approximately the cross-track displacement of 2.83 cm, and that of the MAI phase is equal to the along-track displacement of about 10 m (*n* = 0.5). For the TerraSAR-X system, one fringe of the InSAR and MAI phase correspond to about 1.55 cm and 4.8 m, respectively, and one fringe of the InSAR and MAI phase for the ALOS PALSAR system are approximately 11.8 cm and 8.9 m, respectively. Although the measurement accuracy of the along-track displacement achieved by the MAI technique is much lower than that of the cross-track displacement by the InSAR one, the measurement of the along-track displacement from the MAI phase is very successful because the phase distortions caused by flat-Earth and topographic distortions and atmospheric delay can be removed out as well as is superior to other methods such as the pixel offset method, the incorporation of multiple radar passes, *etc.*

### 2.2. Measurement Uncertainties of MAI Method

The measurement uncertainty of the along-track displacement is calculated as:

(12) $$\sigma_{\Delta x}=\frac{l}{4\pi \cdot n}\sigma_{\phi ,\text{MAI}}$$

where σ_Δ_*_x_* and σ_*ϕ*,*MAI*_ are the standard deviations of the along-track displacement and the MAI phase, respectively. Thus the measurement precision depends on phase noise that is in turn a function of coherence. The interferogram is corrupted by various decorrelations [23] causing coherence to decrease, which results in a deterioration of measurement uncertainty [16].

The standard deviation of MAI phase σ_*ϕ*,*MAI*_ can be defined by using the maximum-likelihood estimator (MLE) as follows [16]:

(13) $$\sigma_{\phi ,\text{MAI}}\approx \frac{1}{\sqrt{N_{L,\text{MAI}}}}\frac{\sqrt{1-\gamma^{2}}}{\gamma },$$

where *N*_*L*,*MAI*_ is the effective number of looks of the MAI interferogram and *γ* is total correlation. The effective number of looks of the MAI interferogram *N*_*L*,*MAI*_ can be defined by the numbers of azimuth looks (*N_a_*) and range looks (*N_r_*), the chirp bandwidth (*B_c_*), the sub-aperture Doppler bandwidth (*B_s_*), the sampling frequency (*f_s_*), the pulse repetition frequency (*PRF*) and the noise reduction factor by an adoptive filtering (*W_f_*) as given by:

(14) $$N_{L,\text{MAI}}=N_{a}\cdot N_{r}\cdot \left(B_{s}/\text{PRF}\right)\cdot \left(B_{c}/f_{s}\right)\cdot W_{f}$$

and the total correlation *γ* can be calculated from forward- and backward-looking interferograms as given by:

(15) $$\gamma =\frac{\gamma_{f}+\gamma_{b}}{2}$$

where γ*_f_* and γ*_b_* are the coherences of the forward- and backward-looking interferograms, respectively. The numbers of azimuth and range looks (*N_a_* and *N_r_*) are determined by using the number of the pixels used for multilooking. The chirp bandwidth (*B_c_*), the sampling time (*t_s_*) and the pulse repetition frequency (*PRF*) are obtained from the system parameters depending on a given SAR system. The sub-aperture Doppler bandwidth (*B_s_*) is defined by [16]:

(16) $$B_{s}=\left(1-n\right)\cdot B_{D}-\Delta f_{DC}\;\left(0.5\leq n<1\right)$$

where *B_D_* is the effective Doppler bandwidth and Δ*f_DC_* is the Doppler centroid difference between the master and slave images. The larger the Doppler centroid difference is, the smaller the sub-aperture Doppler bandwidth is. The noise reduction factor (*W_f_*) indicates how much noise is actually attenuated, and is greater than one when noise indeed reduced. The along-track displacement accuracy can be calculated by the major steps including: (1) generation of a coherence map using Equation (15) from the forward- and backward-looking interferograms on a pixel-by-pixel basis; (2) calculation of the effective number of looks of the MAI interferogram using Equation (14) from system and MAI processing parameters; (3) creation of a MAI phase accuracy map using Equation (13) on a pixel-by-pixel basis; (4) generation of an along-track displacement accuracy map using Equation (12) from the MAI phase accuracy map on a pixel-by-pixel basis. The along-track displacement accuracy map enables us to expect how accurate measurement can be archived in a given interferometric pair.

## 3. Theoretical Accuracies of MAI Measurements

In this paper, we have investigated the expected accuracy of MAI measurements on the basis of the theoretical formula for the existing X-, C- and L-band satellite SAR systems such as the X-band systems of TerraSAR-X, COSMO-SkyMed and Kompsat-5, the C-band systems of ERS-1/2, Envisat, Sentinel-1 and Radarsat-2, and the L-band systems of JERS-1, ALOS PALSAR, ALOS2-PALSAR2. And we have also demonstrated the similarity between the expected and empirical MAI measurement accuracies using X-band COSMO-SkyMed, C-band ERS and L-band ALOS PALSAR interferometric pairs.

### 3.1. X-Band

There are TerraSAR-X, COSMO-SkyMed and Kompsat-5 in the current X-band SAR systems. Their general system parameters are summarized in Table 1. Note that the system parameters are not fixed but changeable. TerraSAR-X is a German Earth-observation satellite which was launched in June 2007 [24]. The satellite is in a near-polar orbit around the Earth, at an altitude of 514 km. Its primary payload is an X-band radar sensor with the antenna wavelength of about 31 mm (carrier frequency: 9.65 GHz), which acquires high-resolution radar images. TerraSAR-X has three main imaging modes: Spotlight, StripMap and ScanSAR. Among the three modes, TerraSAR-X StripMap mode in single polarization is more suitable to the MAI approach because of the short azimuth antenna length of 4.8 m and the large chirp bandwidth of 100 MHz (Table 1). COSMO-SkyMed are Italian Earth-observation satellites which were respectively launched in June and December 2007, October 2008 and November 2010 [25]. The four satellites are in sun-synchronous polar orbits with a 97.9° inclination at an altitude of 619 km. The COSMO-SkyMed satellites have three main imaging modes: Spotlight, StripMap and ScanSAR. The StripMap mode is divided into HIMAGE and PINGPONG modes. The StripMap HIMAGE is most suitable mode to measure along-track displacements using the COSMO-SkyMed MAI due to the short azimuth antenna length of 5.7 m and the large chirp bandwidth of 117 MHz (Table 1). Kompsat-5 is a Korean Earth-observation radar satellite. Its payload is an X-band radar sensor with the carrier frequency of 9.66 GHz. There are three imaging modes: Standard (ST), High-Resolution (HR) and Wide-Swath (WS). The ST mode is more suited to a precise MAI measurement. Although its chirp bandwidth of 73.24 MHz is not relatively large, the antenna length of 4.48 m is very small (Table 1).

**Figure 2.**
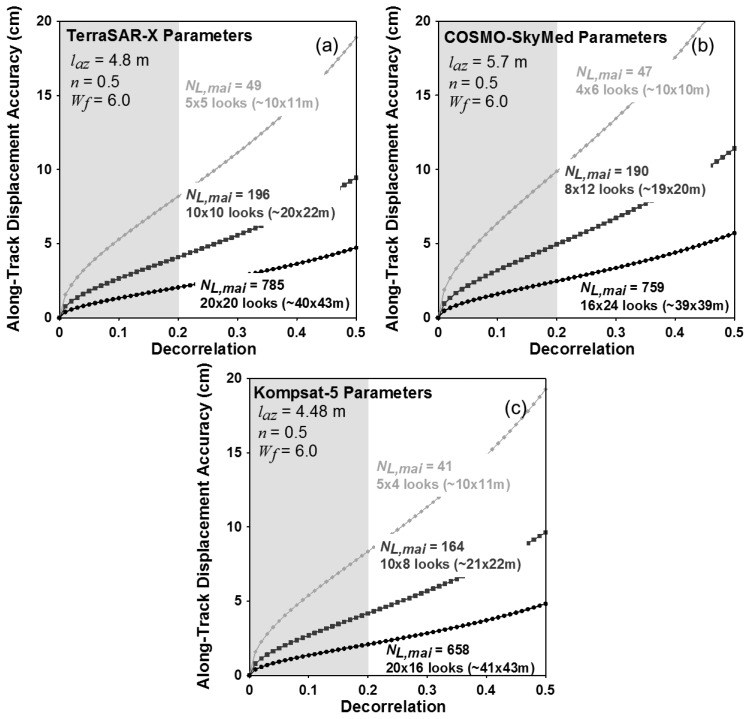
Variation of along-track displacement accuracy with respect to decorrelation for TerraSAR-X, COSMO-SkyMed and Kompsat-5 interferometric pairs.

Figure 2 show variations of theoretical measurement accuracies of the MAI along-track displacement for the X-band SAR systems of TerraSAR-X, COSMO-SkyMed and Kompsat-5, respectively. From Equations (12) to (16), the accuracies are calculated by using the system and processing parameters of the three X-band satellite radar systems as given by Table 1. The numbers of azimuth and range looks are selected to make the azimuth and ground range pixel spacing to be approximately 10 × 10, 20 × 20 and 40 × 40 m, respectively. The incidence angle of 39 deg. is used to calculate the ground range pixel spacing from the range one. The selected numbers of azimuth and range looks are 5 × 5, 10 × 10 and 20 × 20 pixels for TerraSAR-X, respectively, 4 × 6, 8 × 12 and 16 × 24 pixels for COSMO-SkyMed, respectively and 5 × 4, 10 × 8 and 20 × 16 pixels for Kompsat-5, respectively. In this analysis, the normalized squint of 0.5 and the noise reduction factor of 6.0 are used to analyze the MAI theoretical accuracies. As shown in Figures 2, the measurement uncertainties of the MAI along-track displacements rapidly decrease as the total decorrelation decreases and the effective number of looks *N*_*L*,*MAI*_ increases. It means that the MAI measurement performance largely depends on the total decorrelation. When the decorrelation is 0.2, the expected measurement accuracies are about 8.2, 4.1 and 2.0 cm for TerraSAR-X, about 9.9, 4.9 and 2.5 cm for COSMO-SkyMed and 8.3, 4.2 and 2.1 cm for Kompsat-5 in the multilook number of 10 × 10, 20 × 20 and 40 × 40 m, respectively. The current commercial X-band SAR systems are allowed to measure the along-track displacement with the accuracy of about 2.0 cm and ground resolution of about 40 × 40 m using the MAI method when the decorrelation is less than 0.2. The optimal MAI processing [16,17] uses raw data, but TerraSAR-X does not provide them. Thus, the expected accuracies of TerraSAR-X might be reduced.

**Figure 3.**
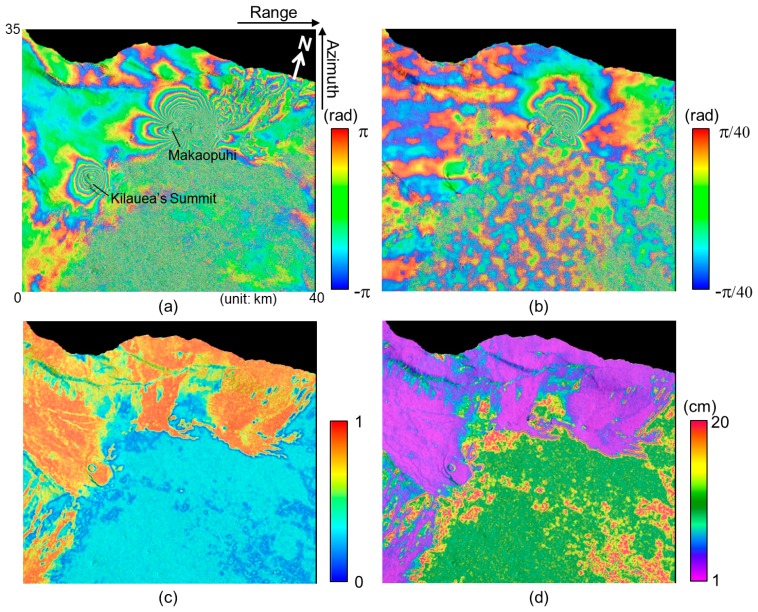
(**a**) InSAR and (**b**) MAI interferograms; (**c**) coherence map and (**d**) the expected MAI displacement accuracy map generated from COSMO-SkyMed interferometric pair.

Figure 3a,b present the InSAR and MAI interferograms generated from the COSMO-SkyMed interferometric pair acquired on 27 February and 7 March 2011. The interferograms show the Kamoamoa fissure eruption which occurred on 5 March 2011 along the east rift zone (ERZ) in Kilauea Volcano, Hawai'i [26]. Thy typical deformation pattern that indicates a dyke opening is seen on the InSAR and MAI interferograms. The InSAR interferogram has been created by a complex multilook operation of 20 × 20 looks (about 42 m × 41 m) in range and azimuth directions. The multilook operation has been applied using a two-step strategy as follows: (1) 5 × 5 looks before the flat-Earth and topographic phase corrections and (2) 4 × 4 looks after the corrections to reduce phase noise [16]. Then the InSAR interferogram has been smoothed by using the Goldstein adaptive filter [27] with a window size of 32 to reduce phase variance.

The MAI interferogram has been created by Jung's MAI approach [16]. For the MAI processing of the interferometric pair, the forward, average and backward Doppler centroids of 591.3 Hz, −51.0 Hz and −683.3 Hz, respectively, and the sub-aperture processing bandwidth of 1217.5 Hz are calculated by MAI azimuth common band filtering [16]. A normalized squint of 0.5, effective antenna length of 5.7 m, PRF of 3360 Hz, effective Doppler bandwidth of 2511 Hz, and Doppler difference of 38.0 Hz, respectively, has been used for this calculation. Forward- and backward-looking SLC images are created from radar raw signal data, and then forward- and backward-looking interferograms are generated, and multilooked and smoothed by the same multilooking factor and filter used for the InSAR processing. The final MAI interferogram is generated after residual flat-Earth and topographic phase corrections are applied [16]. As seen in Figure 3b, there are horizontal stripes due to altitude oscillation of the COSMO-SkyMed satellites. We need to solve the problem to measure precise along-track displacements. However, because it is very hard to perfectly remove the stripes in the MAI interferogram, the expected MAI accuracy might be reduced.

Figure 3c,d show the coherence map and MAI displacement accuracy map, respectively. The coherence map has been created by averaging the forward- and backward-looking interferometric coherence maps. The maps have been calculated with a window size of 5 × 5 pixels in the range and azimuth directions from the forward- and backward-looking interferograms that are multilooked and not filtered. The MAI displacement accuracy map has been generated from the coherence map by using Equations (12) to (16). The minimum value of the accuracies is about 1.1 cm when the coherence is about 0.95. Since the lower part of Figure 3c,d is forest area, the temporal decorrelation is high. Thus in the area, both of the coherence and accuracy are relatively low. The higher part of the coherence and accuracy maps is the region that includes bare rocks formed by lava flow. That is, in the area, high coherences are preserved as well as high measurement accuracies are obtained.

**Figure 4.**
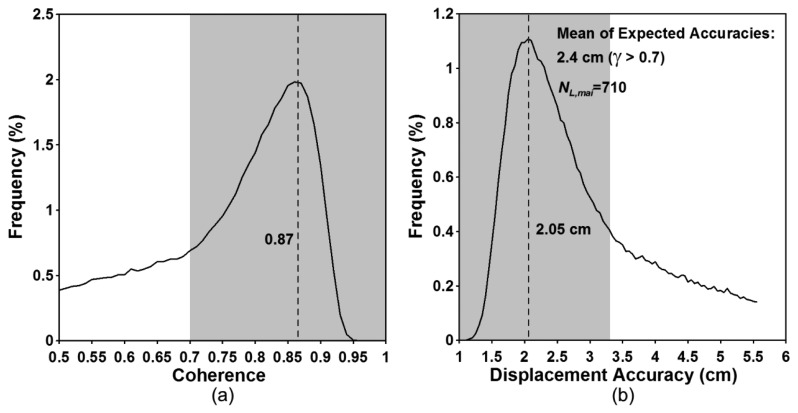
Histograms of (**a**) Interferometric coherence and (**b**) expected accuracy calculated from COSMO-SkyMed MAI interferogram.

Figure 4 show the interferometric coherence histogram and the expected accuracy histogram, respectively. The peak value of the coherence histogram is about 0.87 (Figure 4a) and corresponds to the accuracy of about 2.05 cm (Figure 4b). If we measure along-track displacements in the regions with the coherence of greater than 0.7, the mean of the expected accuracies are about 2.4 cm. The empirical accuracy calculated by using GPS measurements is about 3.09 cm [28]. In the case of COSMO-SkyMed, the expected accuracy is better than the empirical one. It results from the altitude oscillation of the COSMO-SkyMed satellites.

Although the artifact is successfully corrected, the expected MAI accuracy must be reduced because the perfect correction cannot be done. We might need to consider the additional error source of around 0.7 cm in the COSMO-SkyMed X-band MAI measurement.

### 3.2. C-Band

In commercial C-band SAR systems, there are ERS-1/2, Envisat, Radarsat-2 and Sentinel-1. Their general system parameters are summarized in Table 2. It should be noted that the system parameters are not fixed but changeable for Radarsat-2 and Sentinel-1. ERS-1/2 satellites are European remote sensing satellites which were launched in July 1991 and April 1995, respectively [24]. The satellites are in a near-polar orbit at an altitude of 784 km. The payloads are C-band radar sensor with the antenna wavelength of about 56 mm (carrier frequency: 5.30 GHz). ERS-1/2 failed in March 2000 and September 2011. However, the ERS SAR images have been still used for monitoring Earth surface deformations with InSAR method. Envisat satellite is an environmental satellite which was launched in March 2002. The satellite is in a sun-synchronous near-polar orbit at an altitude of 790 km. The Envisat ASAR (advanced synthetic aperture radar) operates in C-band with the radar wavelength of about 56 mm (carrier frequency: 5.33 GHz). The effective azimuth antenna length are 10 m for both of the ERS SAR and Envisat ASAR sensors (Table 2). Radarsat-2 is a Canadian Earth-observation satellite that was successfully launched in December 2007 [29]. It is in sun-synchronous polar orbits at 798 km altitude and has a SAR sensor with multiple polarization modes including a fully polarimetric mode. The Radarsat-2 SAR sensor has three main imaging modes: Single beam mode, ScanSAR and Spotlight. The single beam mode is composed of Standard, Wide, Fine, Multi-look fine, Ultra-fine, Extended high and low, Standard quad polarization and Fine quad polarization. The ultra-fine mode is most suitable mode to measure along-track displacements using the Radarsat-2 MAI due to the short azimuth antenna length of 6.55 m and the large chirp bandwidth of 78.16 MHz (Table 2). Sentinel-1 is a radar imaging satellite mission for land and ocean observations. It is consisting of a constellation of two satellites. Its payload is a C-band SAR with the radar wavelength of 55.5 mm (carrier frequency: 5.40 GHz). There are four imaging modes: SM (StripMap), IW (interferometric wide swath), EW (extra wide swath) and WV (water vapor). The background mode of Sentinel-1 operation is the IW mode, which provides a wide coverage of 250 km with medium resolutions of 5 and 20 m in range and azimuth directions, respectively. A constellation of two Sentinel-1 satellites will provide a revisit of six days. The short temporal baseline will enhance interferometric coherence in general [17,30].

**Figure 5.**
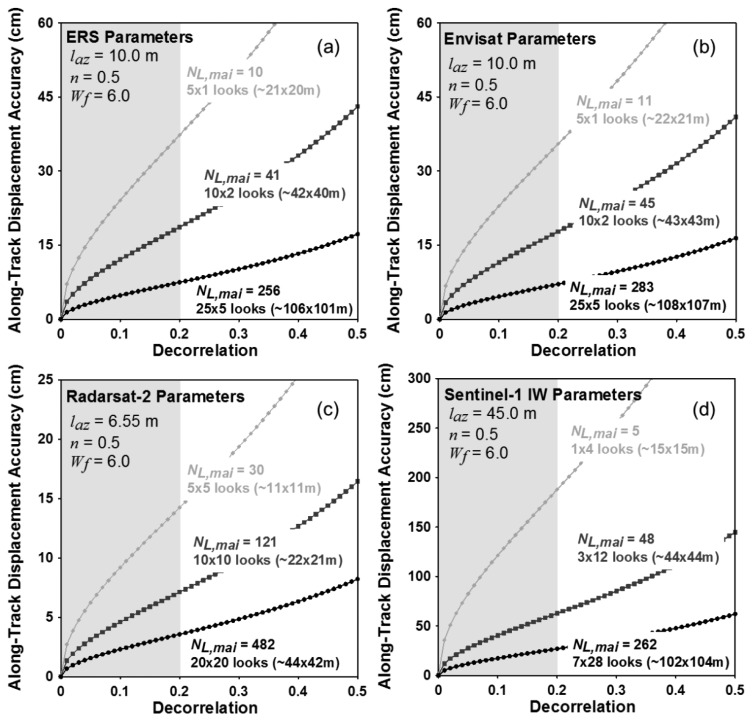
Variation of along-track displacement accuracy with respect to decorrelation for ERS-1/2, Envisat, Radarsat-2 ultra-fine and Sentinel-1 IW interferometric pairs.

Figures 5 present variations of theoretical accuracies of the MAI along-track displacement measurements for C-band SAR systems of ERS-1/2, Envisat, Radarsat-2 and Sentinel-1, respectively. From Equations (12) to (16), the accuracies are calculated by using the system and processing parameters of the four C-band satellite radar systems as given by Table 2.

The numbers of azimuth and range looks are selected to make the azimuth and ground range pixel spacing to be approximately 10 × 10, 40 × 40 and 100 × 100 m for ERS, Envisat and Sentinel-1, respectively and 10 × 10, 20 × 20 and 40 × 40 m for Radarsat-2. The conversion of the ground range pixel spacing from the range one is performed by using the incidence angle of 39 degree for Radarsat-2 and Sentinel-1. The selected numbers of azimuth and range looks are 5 × 1, 10 × 2 and 25 × 5 pixels for ERS-1/2 and Envisat, respectively, 5 × 5, 10 × 10 and 20 × 20 pixels for Radarsat-2 ultra-fine mode, respectively and 1 × 4, 3 × 12 and 7 × 28 pixels for Sentinel-1 IW mode, respectively. The normalized squint of 0.5 and the noise reduction factor of 6.0 are used to analyze the MAI theoretical accuracies. As shown in Figure 5, the measurement uncertainties of the MAI along-track displacements rapidly decrease as the total decorrelation decreases and the effective number of looks *N*_*L*,*MAI*_ increases. It means that the MAI measurement performance largely depends on the total decorrelation. When the decorrelation is 0.2, the expected measurement accuracies are about 37.3, 18.6 and 7.5 cm for ERS-1/2, about 35.5, 17.7 and 7.1 cm for Envisat and 187.9, 62.6 and 26.8 cm for Sentinel-1 IW in the multilook number of 10 × 10, 40 × 40 and 100 × 100 m, respectively, 14.2, 7.1, 3.6 cm for Radarsat-2 ultra-fine in the multilook number of 10 × 10, 20 × 20 and 40 × 40 m, respectively. When the decorrelation is 0.2, the ERS and Envisat systems are allowed to measure the along-track displacement with the accuracy of about 7.0 cm and ground resolution of about 100 × 100 m using the MAI method. The expected accuracy for Sentinel-1 IW is about 26.8 cm in the ground resolution of about 100 × 100 m when the decorrelation is 0.2. Due to a much higher temporal resolution of Sentinel-1, we expect much higher coherence from Sentinel-1 than ERS and Envisat images. Consequently, we expect that more accurate along-track displacements can be measured by the Sentinel-1 MAI method. The expected accuracy for Radarsat-2 ultra-fine is about 3.6 cm in the ground resolution of about 40 × 40 m when coherence is 0.8.

**Figure 6.**
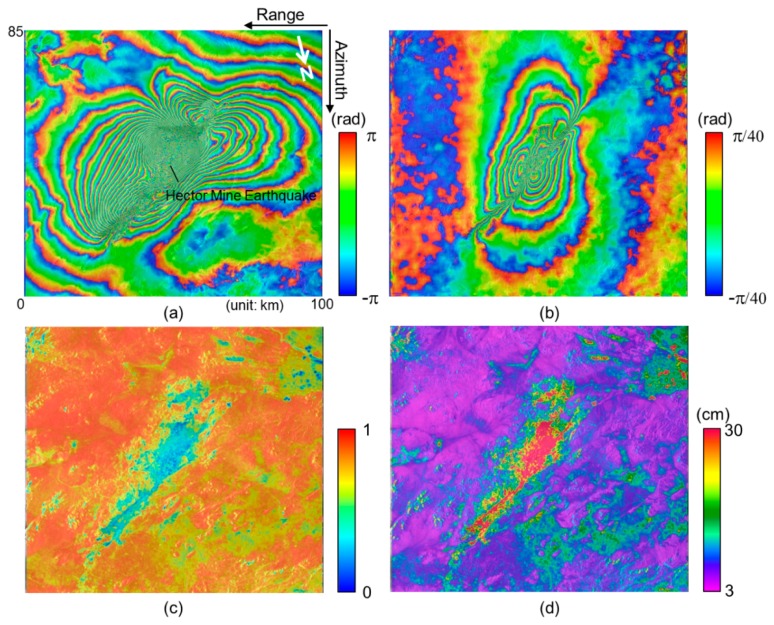
(**a**) InSAR and (**b**) MAI interferograms; (**c**) coherence map and (**d**) the expected MAI displacement accuracy map generated from ERS interferometric pair.

Figure 6a,b present the InSAR and MAI interferograms generated from the ERS-2 interferometric pair acquired on 15 September and 20 October 1999. The interferograms cover the epicenter of the Hector Mine earthquake, California, which occurred on 16 October 1999 [16,17]. The typical deformation pattern that indicates a right-lateral strike-slip fault is seen on the InSAR and MAI interferograms. The InSAR interferogram has been created by a complex multilook operation of 5 × 25 looks (approximately 106 m × 101 m) in range and azimuth directions. The multilook operation has been applied using a two-step strategy as follows: (1) 1 × 5 looks before the flat-Earth and topographic phase corrections and (2) 5 × 5 looks after the corrections to reduce phase noise [16]. Then the InSAR interferogram has been smoothed by using the Goldstein adaptive filter [27] with a window size of 32 to reduce phase variance.

The MAI interferogram has been created by Jung's MAI approach [16]. For the MAI processing of this interferometric pair, the forward, average and backward Doppler centroids of 404.9 Hz, 49.6 Hz and −305.7 Hz, respectively, and the sub-aperture processing bandwidth of 650.8 Hz are calculated by MAI azimuth common band filtering [16]. A normalized squint of 0.5, effective antenna length of 10.0 m, PRF of 1680 Hz, effective Doppler bandwidth of 1361 Hz, and Doppler difference of 59.9 Hz, respectively, has been used for the MAI calculation. Forward- and backward-looking SLC images are created from radar raw signal data, and then forward- and backward-looking interferograms are generated, and multilooked and by the same multilook factor and smoothed by the filter used for the InSAR processing. The final MAI interferogram is generated after residual flat-Earth and topographic phase corrections are applied [16].

Figure 6c,d show coherence map and the MAI displacement accuracy map, respectively. The coherence map has been created by averaging the forward- and backward-looking interferometric coherence maps. The maps have been calculated with a window size of 5 × 5 pixels in the range and azimuth directions from the forward- and backward-looking interferograms that are multilooked and not filtered. The MAI displacement accuracy map has been generated from the coherence map by using Equations (12) to (16). The minimum value of the accuracies is about 2.95 cm when the coherence is about 0.96. Since the center part of Figures 6c,d is a large and complex displacement area, the decorrelation is high. Thus in the area, both of the coherence and accuracy are relatively low. However, there are the apparent fringes at the region as seen in the MAI interferogram of Figure 6b. The rest of the parts on the coherence and accuracy maps are the desert area, so that a high coherence is preserved and a high measurement accuracy is obtained as well. As seen in Figure 6d, the accuracy largely depends on the interferometric coherence. If the coherence of more than 0.9 is calculated from a given interferogram, the MAI measurement accuracy of 5.0 cm is expected.

**Figure 7.**
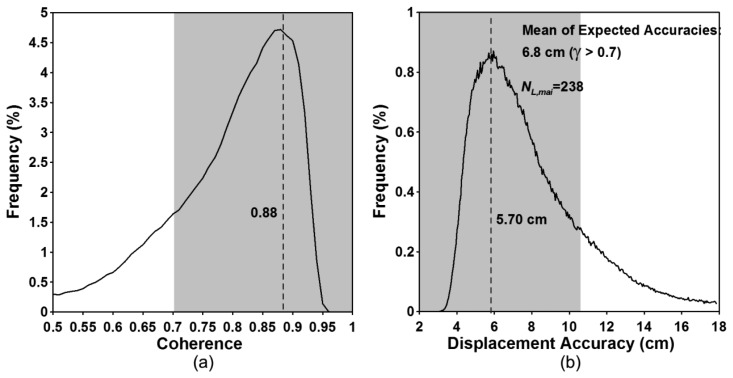
(**a**) Interferometric coherence and (**b**) expected accuracy histograms calculated from ERS MAI interferogram.

Figure 7 show the interferometric coherence histogram and the expected accuracy histogram, respectively. The peak value of the coherence histogram is about 0.88 (Figure 7a) and corresponds to the accuracy of about 5.70 cm (Figure 7b). The pixels having the coherence of more than 0.7, 0.8 and 0.9 are up to 80, 57 and 15 percent of the total pixels, respectively. The coherence values of 0.7, 0.8 and 0.9 correspond to the along-track displacements of 10.5, 7.7 and 5.0 cm, respectively. If we measure along-track displacements in the regions with coherence greater than 0.7, the mean of the expected accuracies are about 6.8 cm. The empirical accuracy calculated by using GPS measurements is about 5.47 cm [17]. In this case, the expected accuracy is lower than the empirical one. In that the former includes some part of the large and complex displacement area in the accuracy calculation, but the latter does not. If we use the coherent pixels of greater than 0.8, the mean of the expected accuracies are about 5.9 cm. The result shows that the difference between the empirical and expected accuracies is small.

### 3.3. L-Band

There are JERS-1, ALOS PALSAR and ALOS2 PALSAR2 in commercial L-band SAR systems. Their general system parameters are summarized in Table 3. The system parameters are changeable for ALOS PALSAR and ALOS2 PALSAR2. JERS-1 satellite is a Japanese remote sensing satellite which was launched in February 1992 and ended in October 1998. The satellite is in a near-polar orbit at an altitude of 568 km. The payload is an L-band radar sensor with the antenna wavelength of about 23.5 cm (carrier frequency: 1.275 GHz). ALOS PALSAR is also a Japanese satellite which was launched in January 2006 and ended in May 2011. The satellite is in a sun synchronous near-polar orbit at an altitude of 691 km. The ALOS PALSAR operates in L-band with the radar wavelength of about 23.62 cm (carrier frequency: 1.27 GHz). Its effective azimuth antenna length are 8.9 m (Table 2).

ALOS2 PALSAR2 is an Earth-observation satellite that was successfully launched in May 2014. It is in sun-synchronous polar orbits at 628 km altitude. The ALOS2 PALSAR2 sensor has three main imaging modes: Strip map, ScanSAR and Spotlight. The StripMap mode is relatively suitable to measure along-track displacements using the PALSAR2 MAI. The effective azimuth antenna dimension and chirp bandwidth are 9.9 m and 84.0 MHz for the PALSAR2 sensor, respectively (Table 2). The antenna length of the PALSAR2 system is slightly larger than that of the PALSAR one, but the chirp bandwidth of the PALSAR2 is three-times larger than that of the PALSAR. Therefore, the PALSAR2 system can be more suitable to the MAI measurement rather than the PALSAR one.

**Figure 8.**
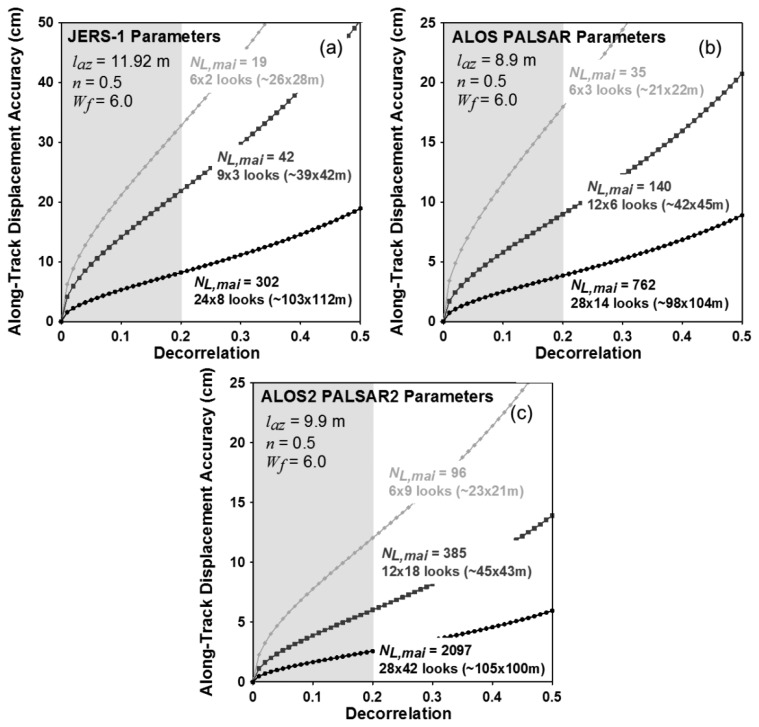
Variation of along-track displacement accuracy with respect to decorrelation for JERS-1, ALOS PALSAR and ALOS2 PALSAR2 interferometric pairs.

Figure 8 show variations of theoretical MAI accuracies for L-band SAR systems of JERS-1, PALSAR and PALSAR2, respectively. From Equations (12) to (16), the accuracies are calculated by using the system and processing parameters of the three L-band satellite radar systems as given by Table 2.

The numbers of azimuth and range looks are selected to make the azimuth and ground range pixel spacing to be approximately 10 × 10, 40 × 40 and 100 × 100 m. The conversion of the ground range pixel spacing from the range one is performed by using the incidence angle of 39 degree for the three L-band SAR systems. The selected numbers of azimuth and range looks are 6 × 2, 9 × 3 and 24 × 8 pixels for JERS-1, respectively, 6 × 3, 12 × 6 and 28 × 14 pixels for ALOS PALSAR FBS mode, respectively and 6 × 9, 12 × 18 and 28 × 42 pixels for ALOS2 PALSAR2 ultrafine single polarization mode, respectively. The normalized squint of 0.5 and the noise reduction factor of 6.0 are used to analyze the MAI theoretical accuracies. The MAI measurement performance largely depends on the total decorrelation like the X- and C-band SAR systems. When the decorrelation is 0.2, the expected measurement accuracies are about 32.7, 21.8 and 8.2 cm for JERS-1, about 18.0, 9.0 and 3.8 cm for ALOS PALSAR and 12.0, 6.0 and 2.6 cm for ALOS2 PALSAR2 in the multilook number of 10 × 10, 40 × 40 and 100 × 100 m, respectively. When the decorrelation is 0.2, the PALSAR and PALSAR2 systems are respectively allowed to measure the along-track displacement with the accuracy of about 4.0 and 2.5 cm in the ground resolution of about 100 × 100 m.

**Figure 9.**
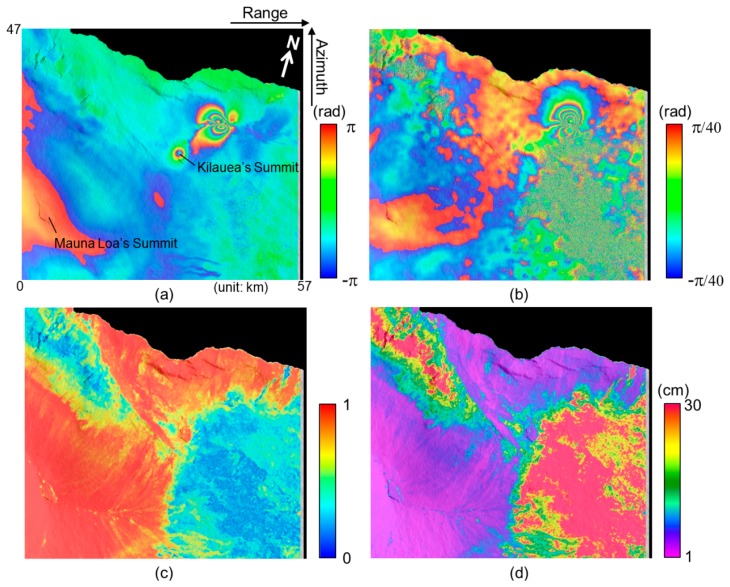
(**a**) InSAR and (**b**) MAI interferograms; (**c**) coherence map and (**d**) the expected MAI displacement accuracy map generated from ALOS PALSAR interferometric pair.

Figure 9a,b present the InSAR and MAI interferograms of the ALOS PALSAR ascending pair acquired on 5 May and 20 June, 2007. The interferograms show the 2007 Father's day intrusion and eruption along the east rift zone (ERZ) in Kilauea Volcano, Hawai'i [4]. Thy typical deformation pattern that indicates a dyke opening is seen on the InSAR and MAI interferograms. The InSAR interferogram has been created by a complex multilook operation of 8 × 16 looks (approximately 60 m × 56 m) in range and azimuth directions. The multilook operation has been applied using a two-step strategy as follows: (1) 1 × 4 looks before the flat-Earth and topographic phase corrections and (2) 4 × 4 looks after the corrections to reduce phase noise [16]. Finally, the InSAR interferogram has been smoothed by using the Goldstein adaptive filter [27] with a window size of 32.

The MAI interferogram has been created by Jung's MAI approach [16]. For the MAI processing of this interferometric pair, the forward, average and backward Doppler centroids of 499.5 Hz, 95.4 Hz and −308.6 Hz, respectively, and the sub-aperture processing bandwidth of 806.5 Hz are calculated by MAI azimuth common band filtering [16]. A normalized squint of 0.5, effective antenna length of 8.9 m, PRF of 2160 Hz, effective Doppler bandwidth of 1614 Hz, and Doppler difference of 3.0 Hz, respectively, has been used for this calculation. Forward- and backward-looking SLC images are created from radar raw signal data, and then forward- and backward-looking interferograms are generated, and multilooked and by the same multilook factor and smoothed by the filter used for the InSAR processing. The final MAI interferogram is generated after residual flat-Earth and topographic phase corrections are applied [16].

Figure 9c,d show the coherence map and the MAI displacement accuracy map, respectively. The minimum value of the expected accuracies is about 1.30 cm when the coherence is about 0.99. Since the lower part of Figure 9c,d is forest area, the temporal decorrelation is high. Thus in the area, both of the coherence and accuracy are relatively low. However, the L-band coherence is much higher than the X-band one (see Figure 3c), because the surface scattering is dominant in the L-band system while the volume scattering is dominant in the X-band system.

The different scattering is caused by the difference between the X- and L-band radar wavelengths. The higher part of the coherence and accuracy maps is the region that includes bare rocks formed by lava flow. That is, in the area, high coherences are preserved, and high measurement accuracies are obtained as well.

**Figure 10.**
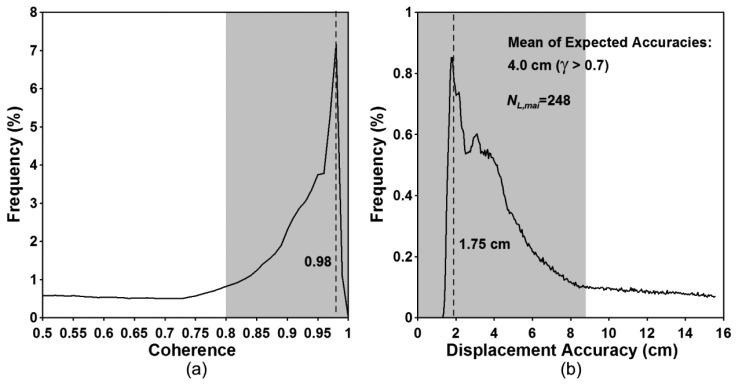
(**a**) Interferometric coherence and (**b**) expected accuracy histograms calculated from ALOS PALSAR MAI interferogram.

Figure 10 show the interferometric coherence histogram and the expected accuracy histogram, respectively. The peak value of the coherence histogram is about 0.98 (Figure 10a) and corresponds to the accuracy of about 1.75 cm (Figure 10b). The pixels having the coherence of more than 0.7, 0.8 and 0.9 are up to 54, 48 and 35 percent of the total pixels, respectively. The coherence values of 0.7, 0.8 and 0.9 correspond to the along-track displacements of 9.2, 6.7 and 4.3 cm, respectively. If we measure along-track displacements in the regions with coherence greater than 0.7, the mean of the expected accuracies are about 4.0 cm. The empirical accuracy calculated by using GPS measurements is about 3.7 cm [4]. The expected accuracy is very similar to the empirical one.

## 4. Discussion and Conclusions

The InSAR technique has been successfully used for the precise measurement of Earth surface displacements. However, the technique can measure only one-dimensional displacements along the radar antenna's line-of-sight (LOS) direction. The measurement of the precise along-track displacements has been made with the multiple aperture SAR interferometry (MAI). The empirical accuracy assessment of the MAI technique has been performed by using ERS and ALOS data, and their empirical accuracies were about 6.3 cm and 3.57 cm, respectively. Even though we know the empirical accuracies, it is very important to estimate the expected MAI measurement accuracies when an interferometric pair is given.

In this study, we have investigated the expected MAI measurement accuracy on the basis of the theoretical formula for the existing X-, C- and L-band satellite SAR systems and shown the similarity between the expected and empirical MAI measurement accuracies. The X-band SAR systems have the expected accuracy of about 2–3 cm with the ground resolution of 40 × 40 m (γ = 0.8), and the L-band PALSAR series have the expected accuracy of about 3–4 cm with the ground resolution of 100 × 100 m (γ = 0.8). For the C-band systems, the expected accuracy achieved from Radarsat-2 high resolution images is about 3–4 cm with 40 × 40 m resolution (γ = 0.8). The ERS-1/2 and Envisat SAR systems have the expected accuracy of about 7 cm with 100 × 100 m resolution. The Sentinel-1 IW systems, which has a small Doppler bandwidth to enlarge the range swath, has about 27 cm with 100 × 100 m resolution. The results show that we can estimate the expected MAI measurement accuracy from a given interferometric pair using the system and processing parameters and coherence.

The comparison of the expected MAI measurement accuracies among X-, C- and L-band systems indicates that the MAI measurement accuracies of the X-band systems are much higher than those of C- or L-band systems because the X-band systems have shorter azimuth antenna lengths. However, if the large temporal baseline is considered, the L-band systems can be much better than X- or C-band ones because of a lower temporal decorrelation. It can be summarized that: (1) for the case of deformation events such as Earthquakes and volcanic eruptions, the X-band systems can be more suitable to measure the precise along-track deformations if the coherence is preserved well and (2) for the case of slow-moving deformations, the L-band systems can be better due to a lower temporal decorrelation. Moreover, multi-temporal InSAR or multi-stacking methods can largely improve the MAI measurement accuracies, especially for the slow-moving deformations.

## Figures and Tables

**Table 1.** System parameters of X-band satellite radar systems.

**Parameters**	**TerraSAR-X ^1^**	**COSMO-SkyMed ^2^**	**Kompsat-5 ^3^**
Effective Azimuth Antenna Dimension (m)	4.8	5.7	4.48
Effective Doppler Bandwidth (Hz)	2770	2670	3110
Pulse Repetition Frequency (Hz)	3800	3000	3530
Chirp Bandwidth (MHz)	100	117	73.24
Carrier Frequency (GHz)	9.65	9.6	9.66
Sampling Frequency (MHz)	109.89	146.25	88.125
Azimuth Pixel Spacing (m)	2.0	2.4	2.1
Ground Range Pixel Spacing (m)	2.2	1.6	2.7

1 TerraSAR-X StripMap mode in single polarization;

2 COSMO-SkyMed StripMap HIMAGE mode and

3 Kompsat-5 StripMap mode.

**Table 2.** System parameters of C-band satellite radar systems.

**Parameters**	**ERS-1/2**	**Envisat**	**Radarsat-2 ^1^**	**Sentinel-1 ^2^**
Effective Azimuth Antenna Dimension (m)	10	10	6.55	40
Effective Doppler Bandwidth (Hz)	1500	1500	2308	380
Pulse Repetition Frequency (Hz)	1680	1650	3637	522
Chirp Bandwidth (MHz)	15.55	16.00	78.16	56.5
Carrier Frequency (GHz)	5.300	5.331	5.405	5.405
Sampling Frequency (MHz)	18.96	18.00	112.68	64.35
Azimuth Pixel Spacing (m)	4.2	4.3	2.2	14.5
Ground Range Pixel Spacing (m)	20.2	21.3	2.1	4.1

1 Radarsat-2 Ultra-Fine mode and

2 Sentinel-1 Interferometric Wide swath mode.

**Table 3.** System parameters of L-band satellite radar systems.

**Parameters**	**JERS-1**	**PALSAR ^1^**	**PALSAR2 ^2^**
Effective Azimuth Antenna Dimension (m)	11.92	8.9	9.9
Effective Doppler Bandwidth (Hz)	1157	1700	1515
Pulse Repetition Frequency (Hz)	1600	2160	2000
Chirp Bandwidth (MHz)	15.0	28.0	84.0
Carrier Frequency (GHz)	1.275	1.27	1.258
Sampling Frequency (MHz)	17.10	32.00	100.0
Azimuth Resolution (m)	4.3	3.5	3.8
Ground Range Resolution (m)	13.9	7.4	2.4

1 ALOS PALSAR FBS (Fine Beam Single Polarization) Mode;

2 ALOS2 PALSAR2 Ultrafine Single Polarization mode.
